# Identification of a 5-Methylcytosine Site that may Regulate C/EBPβ Binding and Determine Tissue-Specific Expression of the *BPI* Gene in Piglets

**DOI:** 10.1038/srep28506

**Published:** 2016-06-24

**Authors:** Li Sun, Jing Wang, Xuemei Yin, Shouyong Sun, Chen Zi, Guoqiang Zhu, Shenglong Wu, Wenbin Bao

**Affiliations:** 1 Key Laboratory for Animal Genetics, Breeding, Reproduction and Molecular Design of Jiangsu Province, College of Animal Science and Technology, Yangzhou University, Yangzhou 225009, P. R. China; 2 College of Veterinary Medicine, Yangzhou University, Yangzhou, Jiangsu, P. R. China

## Abstract

Bactericidal/permeability-increasing protein (BPI) plays an important role in innate immune defense in mammals. A previous study showed that *BPI* gene expression correlates to gram-negative bacteria resistance. However, this gene showed tissue-specific expression in piglets and strongly expressed only in the digestive tract. To investigate the mechanisms governing the tissue-specificity, bisulfite sequencing PCR and next generation sequencing were used for high accuracy methylation quantitation of CpG islands of *BPI* gene upstream in 11 different tissues from weaned Yorkshire piglets. Additionally, qPCR was used to examine mRNA levels of *BPI* gene as well as transcription factor. We additionally analyzed transcriptional regulation by studying key 5-methylcytosine sites and transcription factors. Results showed that *BPI* mRNA levels significantly correlated with the overall methylation as well as methylation at mC-15 which was non-CpG site, no significant correlation could be found between the *BPI* and transcription factor mRNA levels, EMSA test showed that C/EBPβ could interact with *BPI* wild-type promoter DNA, but not methylated DNA. So we confirmed that methylation of mC-15 residue could inhibit the ability of C/EBPβ binding to the *BPI* promoter and affect the expression, and this mechanism probably plays a role in the tissue specificity of *BPI* gene expression in weaned piglets.

Bactericidal/permeability-increasing protein (BPI), an endogenous cationic protein found in humans and other mammals, plays an important role in the innate immune defense. This protein, which is primarily located in the aniline blue particles of polymorphonuclear leukocytes (PMNs), participates in killing gram-negative bacteria and also in neutralizing endotoxin and lipopolysaccharide (LPS)^1^. It also has several other biological roles: it promotes complement activation and opsonization for increased phagocytosis, inhibits angiogenesis and the release of inflammatory mediators, and additionally protects against fungal and protozoan infections^2^. Thus, BPI is an attractive target in the management of both infections and inflammatory diseases, and recombinant BPI and its derivatives have shown promise in the treatment of gram-negative bacterial infections in preclinical as well as clinical trials. The effects of porcine BPI have been well documented: in 1991, Weiss *et al*. found that it played a role in the resistance to *Escherichia coli* (*E. coli*)^3^. An examination of biological activity showed that porcine BPI can kill gram-negative bacteria and neutralize endotoxin^4^. Subsequent *in vitro* assays showed that antibodies to human BPI enhance the biological activity of porcine BPI^5^. In recent years, *BPI* was identified as a candidate gene for disease-resistance breeding in pigs^6^, and Vandermeer *et al*. found that recombinant BPI administration protects pigs against endotoxemia-induced acute lung injury^7^. Additionally, studies from our group have indicated a direct role for the porcine *BPI* gene in preventing *E. coli* F18-induced diarrhea and edema in weaned piglets, and have also shown that resistance to this bacterial strain requires the upregulation of *BPI* gene expression in the gut^8^,^9^.

Epigenetic modifications via DNA methylation regulate gene expression by promoting or inhibiting transcription factor binding to DNA^10^. In higher organisms, DNA methylation is differentially regulated in different tissues or at different developmental stages^11^,^12^. The use of bisulfite sequencing PCR (BSP) in combination with Sanger sequencing has shown that *BPI* gene expression significantly correlates with promoter CpG island methylation in the duodenum of Sutai piglets of different ages^13^. Additionally, we found that *BPI* gene is highly expressed in the digestive tract, but shows only low-level expression in other tissues^8^,^9^. However, the mechanisms governing this tissue-specific expression are unclear, and in this study, we investigated whether they involve promoter methylation.

While the traditional approaches of pyrosequencing or Sanger sequencing are still frequently used for highly targeted analyses involving selected genomic regions, these methods show drawbacks when it comes to hypothesis-driven analyses, as they are limited in their quantitative accuracy, the read length, and sample throughput^14^,^15^,^16^,^17^. With the development of low-cost, high-output next-generation sequencing (NGS), 5-methylcytosine (mC) quantitation methods (for example, MeDIP-seq, MethylCap and reduced representation bisulfite sequencing [RRBS]), are now predominantly used in whole genome sequencing, but the quantitation accuracy of single site still remains low (about 5%). By combining the benefits of bisulfite conversion, targeted amplification, tagmentation-based library construction, and NGS, we have developed a novel method termed bisulfite amplicon sequencing (BSAS) for targeted digital high accuracy quantitation of DNA methylation^18^. In this study, to investigate the genetic mechanisms underlying the tissue-specific expression of the *BPI* gene, we used BSAS to examine the *BPI* gene promoter CpG island methylation, and additionally analyzed the regulation of mRNA expression by examining key 5-mC sites and key transcription factors in 11 different tissues from weaned Yorkshire weaned piglets.

## Materials and Methods

### Ethics statement

The animal study proposal was approved by the Institutional Animal Care and Use Committee (IACUC) of the Yangzhou University Animal Experiments Ethics Committee (permit number: SYXK (Su) IACUC 2012-0029). All experimental procedures were performed in accordance with the Regulations for the Administration of Affairs Concerning Experimental Animals approved by the State Council of the People’s Republic of China.

### Experimental animals and sample collection

Yorkshire piglets (n = 3; 35-days-old) were obtained from a national core pig breeding farm, Changzhou Kangle Farming Co., Ltd. Changzhou, Jiangsu, China. The animals used in this study were healthy, raised under similar conditions, and their birth weight, weaning weight, and body size were similar. This experiment was conducted in the Animal Hospital of Yangzhou University in accordance with the regulations of the Administration of Affairs Concerning Experimental Animals (Ministry of Science and Technology, Beijing, China, revised in June 2012). After sacrifice, the heart, liver, spleen, lung, kidney, stomach, muscle, thymus, lymph node, jejunum, duodenum, testicle, epididymis andmarrow were collected in 1.5-mL nuclease-free Eppendorf tubes, snap-frozen immediately in liquid nitrogen, and then stored at –80 °C until further study.

### DNA bisulfite conversion and bisulfite specific PCR

Genomic DNA was extracted from porcine tissues by standard phenol/chloroform extraction and subjected to bisulfite conversion using the EpiTect bisulfite kit (Qiagen, Valencia, CA, USA), according to the manufacturer’s instructions. Touchdown PCR was used to amplify the bisulfite-treated DNA (BST-DNA). The primer sequences were as follows: forward, 5′-TTTAGTTGTGGTAATTATTTTATAATTTTT-3′ and reverse, 5′-AATTAATCAATACCCCTCACCC-3′, and the amplified fragment length was 406 bp. The 25-μL reaction mixes included 1.5 μL DNA template, 2.5 μL 10× PCR buffer, 2 μL Mg^2+^ (25 mmol/L), 0.5 μL forward primer (10 μM/L), 0.5 μL reverse primer (10 μM/L), 2 μL dNTPs (10 mmol/L), 0.5 μL *Taq* polymerase (5 U/μL) and 15.5 μL water. The following reaction conditions were used: 95 °C for 5 min, then 15 cycles of 95 °C for 15 s, 58 °C for 20 s (reduced by 1 °C every 3 cycles) and 72 °C for 30 s; 35 cycles of 95 °C for 15 s, 53 °C for 20 s and 72 °C for 30 s, with a final extension at 72 °C for 10 min.

### NGS library preparation and sequencing

Indexed libraries were generated using TruSeq DNA PCR-free library preparation technology according to manufacturer’s protocol (Illumina, San Diego, CA, USA). In short, Purified PCR products were quantified using the Qubit 2.0 (Invitrogen, USA). A total of 500 ng DNA was then processed, including end repair, Adenylate 3′ Ends, adaptor ligation. PCR-free libraries were purified using Gnome Size Selector beads (Huijie, Wujiang, China), and eluted off the beads in 10 μl MilQ. Double-stranded libraries were quality checked on a High Sensitivity DNA Agilent chip run on the Agilent 2100 Bioanalyzer (Agilent Technologies) for size and molarity determination and were quantity checked by QPCR using BioRad. Based on these metrics, libraries were diluted to 2 nM in MilQ. Equimolar libraries were pooled in equal volumes for denaturation and dilution in HT1 (Illumina, San Diego, CA, USA) buffer. Briefly, 5 μl of pooled NGS library was mixed with 5 μl 0.2 N NaOH for 5 min, then the library was diluted to 20 pM in HT1 buffer for a final volume of 1 ml. PhiX control libraries (Illumina, San Diego, CA, USA) were used to increase diversity of base calling during sequencing. A volume of 600 μl was loaded onto the reagent cartridge for sequencing. Denatured and diluted libraries were sequenced on the Illumina MiSeq benchtop sequencer with the sequencing-by-synthesis technology per manufacturer’s protocol. Runs were set for ‘Generate FASTQ only’ workflow in Illumina Experiment Manager (Illumina, San Diego, CA, USA). Then, 600-cycle MiSeq v.3 reagent cartridges (Illumina, San Diego, CA, USA) were used to sequence libraries with paired-end, dual-indexing 301 cycles per read (2 × 301). Sequencing run monitoring was achieved through BaseSpace beta (basespace.illumina.com). Data was demultiplexed on the MiSeq instrument automatically, and zipped FASTQ files were generated per sample, per read. Data was accessed either in the run analysis folder locally on the instrument, or through BaseSpace beta.

### NGS data analysis and digital methylation quantitation

The raw sequencing reads were cleaned using in-house scripts to trim sequencing adapters and low quality bases (Q < 20) in the 3′ end and ambiguous bases in both ends. The sequence was indexed by converting all C’s and G’s to T’s and A’s, respectively, i.e., two different reference databases. Bismark was used to map the cleaned reads to each of the two reference databases after converting all C’s to T’s. For each read, an in-house script computed the best of all alignments for the different loci using two different reference databases based on the number of mismatches after realigning the original read and reference sequences. The script also determined the methylation status of each cytosine residue by comparing the bisulfite-converted sequence to the reference sequence. Another in-house script piled reads for each cytosine in the reference genome and counted the numbers of reads that contained methylated and unmethylated cytosines, respectively. Finally the methylation of each CpG site was defined as the fraction of methylated reads to that of methylated and unmethylated reads combined.

### Real-time PCR analysis

RNA was isolated from various tissues of the Yorkshire pigs using the TRIzol reagent (Invitrogen, Carlsbad, CA, USA), according to the manufacturer’s instructions. Single-stranded cDNA was generated using the PrimeScript RT-PCR kit (TaKaRa, Dalian, China), following the manufacturer’s instructions. Real-time quantitative PCR was performed using an ABI Prism 7500 sequence-detection system (Applied Biosystems, Foster City, CA, USA) with SYBR Green PCR Master Mix (TaKaRa), according to the manufacturer’s instructions. The *BPI* fragment was amplified using the primers listed in Table 1. *GAPDH, TBP1*, and *ACTB* were used as internal controls; the expression of *BPI* in each sample was normalized to their geometric mean. Triplicate PCR amplifications were performed for each sample. The real-time PCR results were processed using the 2^−ΔΔCt^ method (ΔCt = mean *BPI* expression − mean internal control expression). The ΔCt for liver tissue was arbitrarily defined as 1.0 for relative quantification of the expression levels of this gene in other samples (ΔΔCt = ΔCt of each sample −ΔCt of the liver in each experiment).

### Electrophoretic mobility shift assay (EMSA)

The *BPI* wt (wild-type) probe (Forward primer: AACCCACAACCTCATGGTTC, Reverse primer: GAACCATGAGGTTGTGGGTT) and *BPI* mut (mutant-type) probe (Forward primer: AACCGACTAGTCTATGGTTC, Reverse primer: GAACCATAGACTAGTCGGTT) were generated in *BPI* promoter region for Electrophoretic Mobility Shift Assay (EMSA). All probes were synthesized directly by the Genecreate (Wuhan, China). The probes were treated by CpG methyl transferase *M.Sss*I (#M0226V, 50 units) purchased from New England Biolabs (NEB, America). Whereafter, probes were labeled with biotin-dUTP. Nuclear protein from duodenal tissues was extracted using the nuclear protein extraction kit (Beyotime, China). Electrophoretic mobility shift assays (EMSAs) were performed according to the manufacturer’s instructions of EMSA kit (Thermo Fisher). The binding buffer contained 20 mM Tris-Cl (pH 7.6), 50 mM KCl, 5 mM MgCl, 0.5 mM EDTA, 10% glycerol, 1 mM dithiothreitol (DTT). 10 μl EMSA reaction system contained 2 μl binding buffer, 2 μl nuclear protein, 2 μl labeled probe, 4 μl ddH_2_O. Antibody supershift assay: C/EBPβ antibodies (23431-1-AP) were obtained from ProteinTech (Wuhan, China). C/EBPβ antibodies (1 μl) were added to EMSA reaction mixtures for 1 h at 4 °C prior to addition of the labeled probe. Products were electrophoresed on a non-denaturing 5% polyacrylamide gel. After electrophores, DNAs were transferred to a positive nylon membrane, UV cross-linked, probed with streptavidin-HRP conjugate and incubated with the substrates of the ECL kit.

## Results

### Validation of the CpG Island Fragment Amplification

**Figure 1:**
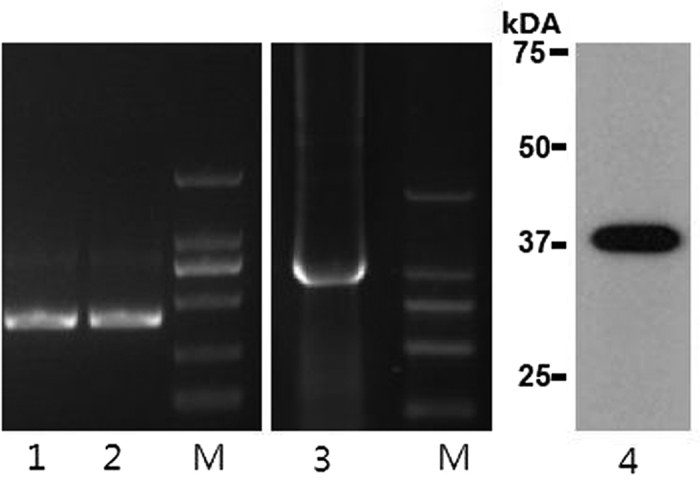
Electrophoretogram. Lanes 1–2: *BPI* gene promoter CpG island PCR products, 406 bp; Lanes 3: *BPI* gene CDS PCR products, 1452 bp; Lanes 4: BPI recombination protein, 38 kDa; M: DL2000 markers.

*BPI* gene promoter CpG island PCR products and CDS PCR products were examined by 1% agarose gel electrophoresis. BPI recombination protein were examined by SDS-PAGE. These size of the amplified fragments corresponded with the expected product sizes (406 bp, 1452 bp and 38 kDa), and each amplification yielded a single specific product that could be used for next experiment (Fig. 1).

### Sequencing quality control

After sequencing, a total of 3.92 million raw reads were generated for 42 samples, out of which 2.32 million reads were mapped after read merging, quality trimming, and elimination of PhiX control reads. The average number of mapped reads was 50,950,000 (10,880,000–126,840,000) for each sample.

### Methylation levels

**Figure 2:**
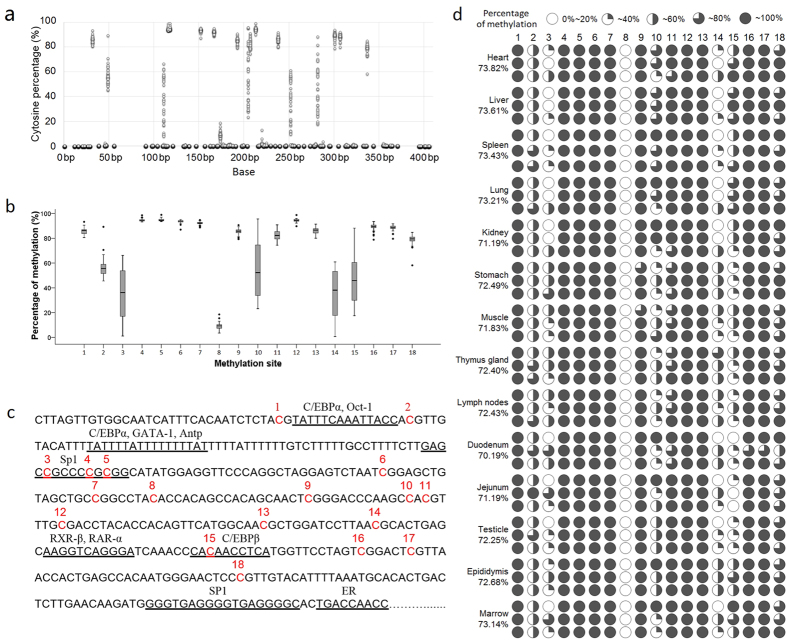
Frequency of DNA methylation of 5-mC sites in different samples. (**a**) Frequency of cytosine of all cytosine sites in the PCR amplicon (406 bp). (**b**) Distribution of DNA methylation range of 5-mC sites in different samples, a total of 18 sites were methylated, and the levels of dispersion of mC-3, mC-10, mC-14, and mC-15 were high in different samples. (**c**) Sequence of the PCR amplicon with the sites of methylation indicated (Sus scrofa10.2:chr17:46796069-46796474), a total of 12 putative transcription factors were identified, two of which (Sp1 and C/EBPβ) contained methylation sites. (**d**) Frequency of methylation of 5-mC sites in different tissues, while the overall methylation levels across different tissues were relatively similar (70.19–73.82%), the level in the duodenum was significant higher than that in the heart, liver, spleen, and lungs (*P* < 0.05).

After sequencing, filtering out of low quality reads, and mapping, the overall percentage of cytosine is shown in Fig. 2a. The sites for which the cytosine ratio was greater than 5% were selected for further analysis (Fig. 2b), and then we found that a total of 18 sites were methylated. In different tissues, the levels of dispersion of mC-3, mC-10, mC-14, and mC-15 were high.

Sequencing of the PCR amplicon revealed 15 CpG sites (Fig. 2c), all of which were methylated. In addition, three non-CpG sites were also found to be methylated (mC-8, mC-10, and mC-15). Additionally, a total of 12 putative transcription factors were identified, two of which (Sp1 [specificity protein 1] and C/EBPβ [CCAAT/enhancer binding protein β]) contained methylation sites.

The methylation levels in different tissues and different sites are shown in Fig. 2d. While the overall methylation levels across different tissues were relatively similar (70.19–73.82%), the level in the duodenum was significant higher than that in the heart, liver, spleen, and lungs (*P* < 0.05).

### Correlation between the methylation level and mRNA Expression

**Figure 3:**
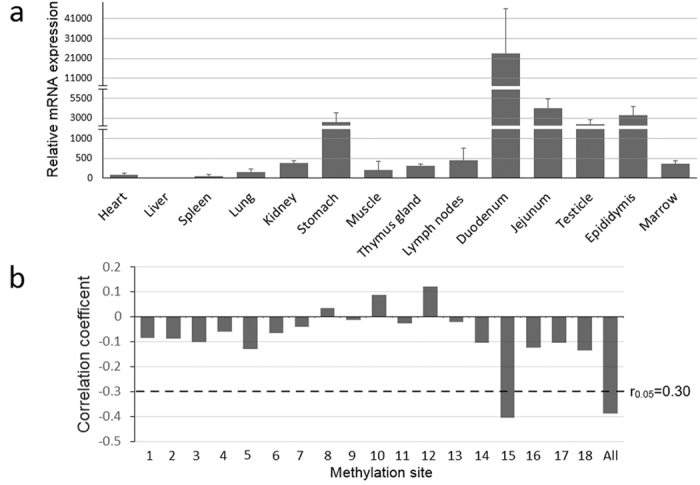
*BPI* gene mRNA expression and the correlation with methylation level. (**a**) *BPI* mRNA expression in different tissues, the expression levels in stomach, jejunum, duodenum, testicle and epididymis were significantly elevated compared to other tissues. (**b**) Pearson’s correlation coefficient for correlations between mRNA expression and methylation level, dashed lines represent the correlation coefficient threshold, mRNA levels significantly correlated with the overall methylation level as well as methylation at the mC-15 site.

The expression of *BPI* gene (as determined by the mRNA level) was significantly elevated in the stomach, jejunum, and duodenum, compared to that in the other tissues examined (*P* < 0.05; Fig. 3a). Pearson correlation analysis showed that the methylation status of the CpG island was negatively correlated with *BPI* mRNA level (Fig. 3b, R = −0.38, r_0.05_ = 0.30, r_0.05_ is the correlation coefficient threshold), with significant correlation coefficients being obtained for only the mC-15 site (R = −0.41).

### Correlation between *BPI* and C/EBPβ mRNA Expression

**Figure 4:**
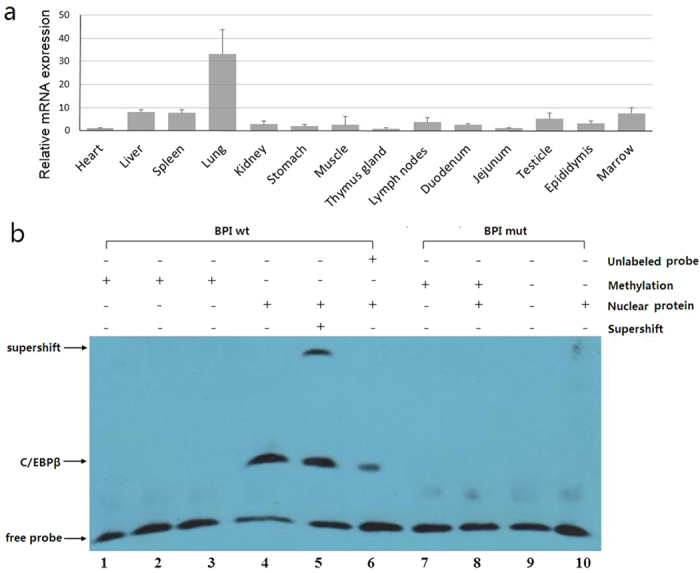
C/EBPβ mRNA expression in different tissues and EMSA analysis of C/EBPβ binding to the *BPI* promoter. (**a**) C/EBPβ mRNA expression in different tissues, C/EBPβ was only highly expressed in the lungs. (**b**) Nuclear proteins from duodenal tissues expressing C/EBPβ were extracted using the nuclear protein extraction kit. After addition of biotin-labeled probe (*BPI* wt, lane 1 to 5; *BPI* mut, lane 7 to 9), C/EBPβ antibody (lane 5) and incubation at RT for 30 min, DNA-protein complexes were analyzed by EMSA with 5% polyacrylamide gel.

We next examined whether there was a correlation between expression of the transcription factors of the C/EBPβ and that of *BPI*; C/EBPβ were only highly expressed in the lungs (Fig. 4a), and no significant correlation could be found when examining the *BPI* and C/EBPβ mRNA levels (R = −0.13).

### EMSA analysis of C/EBPβ binding to the *BPI* gene promoter

To study whether *BPI* methylation could affect the ability of C/EBPβ binding to the *BPI* promoter, we performed EMSA experiments (Fig. 4b). Several shift bands were observed with unmethylated *BPI* wild-type probes (lane 4 to 6), while no shift bands was detected in methylated wild-type probes (lane 1 to 3) and mutant-type probes (lane 7 to 10). This result showed that nuclear extracts from duodenal tissues could interact with *BPI* wild-type probes. When we added C/EBPβ antibody, a strong supershifted band was observed with the unmethylated wild-type probe only (lane 5). This result further indicated C/EBPβ from nuclear extracts could bind specifically to the unmethylated *BPI* wild-type probe.

## Discussion

Given its ability to neutralize endotoxin and protect against gram-negative bacteria^19^,^20^, the BPI has wide application prospects, and can be considered a “super antibiotic”. Porcine BPI also has these functions^6^. Diarrhea and edema, which commonly develop following *E.coli* F18 infection, are two major causes of mortality in postweaning piglets, and these contribute to immense financial losses in the swine-rearing industry. Following infection in pigelets, this bacterium relies on its fimbriae to adhere to the surface of epithelial cells in the small intestine and to bind to the F18 receptor on porcine small intestinal epithelial brush cells. Then, the bacterium attaches, reproduces, and produces enterotoxin, following which symptoms develop in the piglets^21^. In this study, we have reconfirmed the findings from studies done in Sutai piglets, that *BPI* gene mRNA levels are higher in in the stomach, jejunum, duodenum, testicle, epididymis and marrow than in other tissues^8^,^9^. Together, these studies show that the *BPI* gene also shows similar tissue-specific expression in a different breed of weaned piglets. Given that BPI can directly kill *E. coli* F18 and other gram-negative bacteria, and that up-regulated expression is closely related to resistance to intestinal *E. coli* F18, investigating the genetic mechanisms govering *BPI* gene tissue specificity has important implications in biological engineering measures to harness the activity of porcine endogenous BPI protein.

Epigenetic regulation via DNA methylation plays an essential role in the control of biological processes by modulating gene expression. Importantly, DNA methylation is regulated both at the tissue level and also based on the developmental stage^22^,^23^. The traditional methods for quantitative detection of methylation utilize Sanger sequencing and pyrosequencing. Due to the limited number of clones, the quantitative precision is not high enough in the Sanger sequencing method, and additionally, it is also time-consuming^24^; In pyrosequencing, methylation level is determined based on the fluorescence intensity, and hence this method also has limited accuracy^25^. Currently, using Illumina MiSeq sequencing, the read length can be increased to 300 bp × 2, thus covering most of the CpG islands^26^. In this study, which utilized the BSAS method^18^, the average mapped reads for each sample reached 52,260, and this is equivalent to 52,260 clones that were selected in Sanger sequencing, allowing the accuracy to reach 10^−5^; additionally, there is no bias in BSAS, and this ensures accuracy in quantifying the methylation level. In summary, BSAS is superior to other methods. The combination of BSP+ Sanger has been previously used to examine *BPI* gene promoter CpG island methylation^13^. As shown in Fig. 2a, every cytosine site had a certain proportion of cytosine residues, but the proportion was generally less than 1%. This could be attributable to the bisulfite treatment not being complete or to sequencing errrors. After screening, we identified 18 sites where the cytosine proportion was higher than 5%, and these were selected for further analysis. Although the level of methylation was relatively similar in the different tissues, the degree of methylation in the duodenum was significantly lower than that of the heart, liver, spleen and lung; pearson correlation analysis showed that the methylation status of the CpG islands was negatively correlated with *BPI* mRNA expression (*P* < 0.05), suggesting that increased methylation was associated with decreased mRNA expression^27^.

Analysis of the amplified sequence (Fig. 2c) revealed that the 18 methylated sites included all the CpG sites in this sequence, as well as three non-CpG sites. The methylation of non-CpG sites has been found in the embryo, and shows a high correlation with gene expression^28^. DNA methylation can regulate gene transcription and expression by inhibiting the ability of methylation-sensitive transcription factors to bind to DNA or binding repressor proteins to inhibit the binding of methylation non-sensitive transcription factors^29^. Out of the 12 potential transcription factor-binding sites in the PCR amplicon, only the SP1 and C/EBPβ binding sites had methylation sites (mC-3, mC-4, mC-5. and mC-15). Importantly, the mC-15 site that was significantly negatively correlated with *BPI* expression level was located within the C/EBPβ binding site. An experiment by Lennartsson *et al*., where transient transfection of C/EBPα or C/EBPε into HeLa cells resulted in increased promoter activity, indicated a direct or indirect role for C/EBP in regulating BPI expression^30^; Lennartsson *et al*. have also reported that all-trans retinoic acid-induced expression of BPI protein in human myeloid cells correlates with binding of C/EBPβ and C/EBPε to the *BPI* promoter^31^; Miyuki *et al*. have demonstrated the requirement for C/EBPε in mediating *BPI* gene expression in myeloid cells, both in *vitro* and *in vivo*^32^; These studies indicate that members of the C/EBP transcription factor family play an important role in the regulation of *BPI* gene expression. However, there is no significant correlation could be found when examining the *BPI* and *C/EBPβ* mRNA levels. Based on these, we speculate that the methylation at the mC-15 site inhibits the DNA-binding of C/EBPβ and affects *BPI* gene expression^33^, and the EMSA test confirmed that C/EBPβ could interact with *BPI* promoter DNA, but not methylated DNA. So we confirmed that methylation of mC-15 residue could reduce the ability of C/EBPβ to bind the *BPI* promoter and affect the expression, so this mechanism may play a role in the tissue-specific expression of the *BPI* gene.

It is reported that C/EBPβ family members are the major inflammatory mediators in the gastrointestinal tract of infected macaques; indeed, *C/EBPβ* gene expression is increased significantly in macaques infected with simian immunodeficiency virus (SIV) or in animals suffering from chronic diarrhea, compared with controls^34^. Chromatin immunoprecipitation experiments confirmed that a delicate balance of interaction between p65, C/EBP, and trans-activator of transcription can dictate the level of human immunodeficiency virus type 1 transcription in intestinal lamina propria cells^35^. Following exposure of normal tissues to LPS, interleukin-6 (IL-6), interleukin-1 (IL-1), or interferons the *C/EBPβ* expression is increased significantly^36^, indicating that the C/EBPβ can regulate gene expression related to immune, inflammatory, and damage-linked processes^37^,^38^,^39^,^40^. BPI plays an important role in the innate immune response, and our findings indicate that the high expression of *BPI* gene may be related to C/EBPβ in the intestine, where there is high level exposure to gram-negative bacteria on a daily basis.

In summary, the methylation status of *BPI* gene promoter CpG island mC-15 site could affect the binding of the C/EBPβ transcription factor to the *BPI* promoter region in weaned piglets, thereby regulating *BPI* gene expression and resulting in a tissue-specific expression.

## Additional Information

**How to cite this article**: Sun, L. *et al*. Identification of a 5-Methylcytosine Site that may Regulate C/EBPβ Binding and Determine Tissue-Specific Expression of the BPI Gene in Piglets. *Sci. Rep.* **6**, 28506; doi: 10.1038/srep28506 (2016).

## Figures and Tables

**Table 1:** Primers used for real-time PCR (RT-PCR).

Primer name	Sequence of primer
*BPI* RT-PCR primer	F: ATATCGAATCTGCGCTCCGA
	R: TTGATGCCAACCATTCTGTCC
*C/EBPβ* RT-PCR primer	GTCCAAACCAACCGCACAT
	GAAACAACCCCGTAGGAACAT
*GAPDH* RT-PCR primer	ACATCATCCCTGCTTCTACTGG
	CTCGGACGCCTGCTTCAC
*TBP1* RT-PCR primer	AACAGTTCAGTAGTTATGAGC
	AGATGTTCTCAAACGCTTCG
*ACTB* RT-PCR primer	CAGGTCATCACCATCGGCA
	CGTCGCACTTCATGATGGAGT

*BPI* gene and *C/EBPβ* were identified by real-time PCR. The housekeeping genes, *GAPDH*, *TBP1* and *ACTB* were used as the internal controls. The data were analyzed by the cycle threshold (C(t)) method.
