# Corrigendum: Identification of a 5-Methylcytosine Site that may Regulate C/EBPβ Binding and Determine Tissue-Specific Expression of the *BPI* Gene in Piglets

**DOI:** 10.1038/srep33772

**Published:** 2016-12-08

**Authors:** Li Sun, Jing Wang, Xuemei Yin, Shouyong Sun, Chen Zi, Guoqiang Zhu, Shenglong Wu, Wenbin Bao

Scientific Reports 6: Article number: 28506; 10.1038/srep28506 published online: 06 24 2016; updated: 12 08 2016.

In this Article the Authors incorrectly stated that they had developed a novel method termed bisulfite amplicon sequencing (BSAS). The method was reported by Masser et al. (reference 18 in the Article). Thus the following sentence (which appears verbatim in ref 18):

“By combining the benefits of bisulfite conversion, targeted amplification, tagmentation-based library construction, and NGS, we have developed a novel method termed bisulfite amplicon sequencing (BSAS) for targeted digital high accuracy quantitation of DNA methylation^18^”.

should read:

“In a previous study, Masser et al. developed a method termed bisulfite amplicon sequencing (BSAS) for targeted digital high accuracy quantitation of DNA methylation by combining the benefits of bisulfite conversion, targeted amplification, tagmentation-based library construction, and NGS^18^”.

The following sections in “Materials and Methods” failed to appropriately cite the protocols followed:

“DNA bisulfite conversion and bisulfite specific PCR”.

should read:

“DNA bisulfite conversion and bisulfite specific PCR^13^”.

“NGS library preparation and sequencing”.

should read:

“NGS library preparation and sequencing^18^”.

“Real-time PCR analysis”.

should read:

“Real-time PCR analysis^13^”.

An additional reference is listed below as reference 1 and should appear in the text as below:

“NGS data analysis and digital methylation quantitation”.

should read:

“NGS data analysis and digital methylation quantitation^1^”.

The Authors apologize for these errors.
